# Relationship between Expression of Onco-Related miRNAs and the Endoscopic Appearance of Colorectal Tumors

**DOI:** 10.3390/ijms16011526

**Published:** 2015-01-09

**Authors:** Yoshihito Nakagawa, Yukihiro Akao, Kohei Taniguchi, Akemi Kamatani, Tomomitsu Tahara, Toshiaki Kamano, Naoko Nakano, Naruomi Komura, Hirokazu Ikuno, Takafumi Ohmori, Yasutaka Jodai, Masahiro Miyata, Mistuo Nagasaka, Tomoyuki Shibata, Naoki Ohmiya, Ichiro Hirata

**Affiliations:** 1 Department of Gastroenterology, School of Medicine, Fujita Health University, Kutsukake-cho, Aichi 470-1192, Japan; E-Mails: akemie80_82@yahoo.co.jp (A.K.); ttahara@fujita-hu.ac.jp (T.T.); tkamano@fujita-hu.ac.jp (T.K.); naokomaruyama-gi@umin.ac.jp (N.N.); n-kom@hotmail.co.jp (N.K.); h-ikuno@fujita-hu.ac.jp (H.I.); takafumi@fujita-hu.ac.jp (T.O.); 2080319yj@gmail.com (Y.J.); qqur5wd9k@comet.ocn.ne.jp (M.M.); nmitsu@fujita-hu.ac.jp (M.N.); shibat03@fujita-hu.ac.jp (T.S.); nohmiya@fujita-hu.ac.jp (N.O.); ihirata@fujita-hu.ac.jp (I.H.); 2 The United Graduate School of Drug Discovery and Medical Information Sciences, Gifu University, Gifu 501-1193, Japan; E-Mails: yakao@gifu-u.ac.jp (Y.A.); sur144@poh.osaka-med.ac.jp (K.T.)

**Keywords:** microRNA, endoscopic appearance, colorectal tumor

## Abstract

Accumulating data indicates that certain microRNAs (miRNAs or miRs) are differently expressed in samples of tumors and paired non-tumorous samples taken from the same patients with colorectal tumors. We examined the expression of onco-related miRNAs in 131 sporadic exophytic adenomas or early cancers and in 52 sporadic flat elevated adenomas or early cancers to clarify the relationship between the expression of the miRNAs and the endoscopic morphological appearance of the colorectal tumors. The expression levels of miR-143, -145, and -34a were significantly reduced in most of the exophytic tumors compared with those in the flat elevated ones. In type 2 cancers, the miRNA expression profile was very similar to that of the exophytic tumors. The expression levels of miR-7 and -21 were significantly up-regulated in some flat elevated adenomas compared with those in exophytic adenomas. In contrast, in most of the miR-143 and -145 down-regulated cases of the adenoma-carcinoma sequence and in some of the *de novo* types of carcinoma, the up-regulation of oncogenic miR-7 and/or -21 contributed to the triggering mechanism leading to the carcinogenetic process. These findings indicated that the expression of onco-related miRNA was associated with the morphological appearance of colorectal tumors.

## 1. Introduction

MicroRNAs (miRNAs or miRs) are endogenous ~22-nt non-coding RNAs that negatively regulate gene expression by inhibiting the translation of mRNAs in a sequence-specific manner [1,2,3]. More than 2000 miRNAs in the human genome have already been identified [4], and up to one-third of all human mRNAs are predicted to target plural target mRNAs in various cancers [3]. Each miRNA can target more than 200 different transcripts directly or indirectly [5,6], and more than one miRNA can converge on a single mRNA target [3,7]. Therefore, the potential regulatory circuitry afforded by miRNAs is enormous. These findings support the notion that alterations of miRNA copy number and their regulatory genes should be highly prevalent in cancer, because genomic aberrations are closely associated with carcinogenesis. Recent increasing evidence shows that the expression of miRNA genes is deregulated in human cancers [8,9]. Among the tumor-associated miRNAs, miR-143 and -145 are well established as being tumor suppressor miRNAs [10,11]. Since they are transcribed at chromosome position 5q33 as the same primary non-coding RNA (NCR143/145), they are concomitantly down-regulated in most cancers [12]. Previously, we reported that miR-143 and -145 are down-regulated in colon adenomas as well as in cancers [13]. In the current study, we focused on other onco-related miRNAs such as miR-34a [13,14,15], miR-21 [13,16,17,18], and miR-7 [13,19,20] in colorectal cancers, because their levels in colon cancer cells are frequently dysregulated [10,13,14,15,16,17,18,19,20].

**Figure 1:**
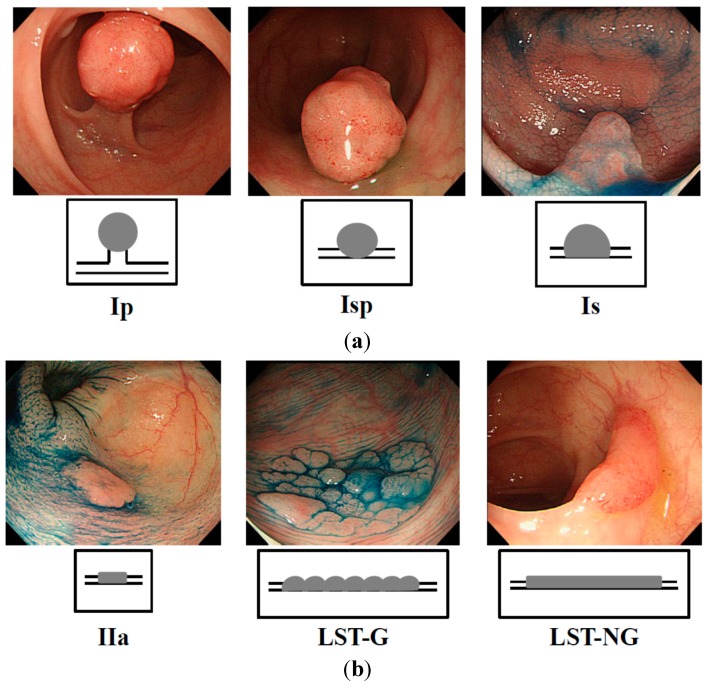
Endoscopic appearance of exophytic and flat elevated tumors (Japanese classification of colorectal carcinoma (8th ed.)) [30]. (**a**) Exophytic type: Ip = pedunculated type; Isp = semipedunculated type; Is = sessile type; and (**b**) Flat elevated type: IIa = superficial elevated type; LST-G = laterally spreading tumor, granular type; LST-NG = laterally spreading tumor, non-granular type.

Recent studies indicated that exophytic tumors (Figure 1a) and flat elevated tumors (Figure 1b) differ in the expression profile of their genome [21,22,23,24,25]. A *Ki-Ras* gene point mutation is frequently observed in exophytic tumors but is very rare in the flat elevated ones [21,22]. Moreover, large flat elevated tumors (over 10 mm), called laterally spreading tumor (LSTs) [25,26] are classified as granular type (LST-G) and non-granular type (LST-NG), according to their endoscopic appearance [25,26,27]. The LST-NG is more malignant than the LST-G [27]. Although the frequency of the *Ki-Ras* point mutation in LST-NG is lower than that in LST-G [28,29], there has been no report as of yet indicating any difference in the miRNA expression profile between exophytic tumors and flat elevated ones.

In the current study, we demonstrate a difference in the miRNA expression profiles between exophytic and flat elevated tumors in patients bearing colorectal tumors.

## 2. Results

### 2.1. Expression of miR -143, -145, -7, -34a, and -21 in Human Colorectal Tumor Tissues

We firstly analyzed the expression levels of miRs-143, -145, -7, -21, and -34a by performing real-time PCR on 135 colorectal cancer samples and 139 colorectal adenomas (Table 1). In this study, there was no significant relationship between the expression profile of these miRNAs and the parameters regarding the size of tumor, site of tumor, sex or age in adenoma or cancer samples (data not shown). Furthermore, there was no significant relationship between the expression profile of these miRNAs and the clinical stage of colorectal cancer or lymph node metastasis (Table 2).

**Table 1:** Expression of miRs-143, -145, -7, -34a, and -21 in human colorectal tumor tissues were evaluated by performing TaqMan® Real-time PCR assays. The relative expression levels were calculated by the ΔΔ*C*_t_ method, with RNU6B used as a control. The relative expression level in normal tissue was indicated as “1”. The expression levels in tumors were designated as down-regulated when the fold change from the expression in the non-tumorous tissue was 0.67 or as up-regulated when the fold change from the expression in the non-tumorous tissue was 1.50. Statistical differences in miRNA levels were evaluated by using Pearson’s χ^2^ test or Fisher’s extract test for differences between two groups. A *p*-value of 0.05 was considered to be significant; statistical analysis was done in this manner for all subsequent Tables.

Colorectal Tumor	*n*	miR-143 ↓	miR-145 ↓	miR-7 ↑	miR-21 ↑	miR-34a ↓
cancer	135	95	99	94	64	74
adenoma	139	94	95	41	59	48
*p* value		0.6234	0.3639	<0.0001	0.4091	0.0007

Down arrow: down-regualted; Up arrow: up-regulated. The red font means that there is a statistical significance.

**Table 2:** Expression of miR -143, -145, -7, -34a, and -21 in human colorectal cancer tissues as evaluated by performing TaqMan^®^ Real-time PCR assays (clinical stage and Dukes’ system).

Colorectal Cancer	*n*	miR-143 ↓	miR-145 ↓	miR-7 ↑	miR-21 ↑	miR-34a ↓
clinical stage (Japanese Classificasion)						
0	27	20	21	12	14	16
I	31	23	23	22	19	11
II	31	18	20	27	11	21
IIIa	28	20	20	19	12	16
IIIb	7	6	7	4	2	5
IV	11	8	8	10	6	5
*p* value		0.6262	0.5328	0.0084	0.3230	0.1448
Dukes’s system						
A or B	92	63	66	63	45	49
C	43	32	33	31	19	25
*p* value		0.4813	0.5401	0.6705	0.6083	0.5957

Down arrow: down-regualted; Up arrow: up-regulated.

**Figure 2:**
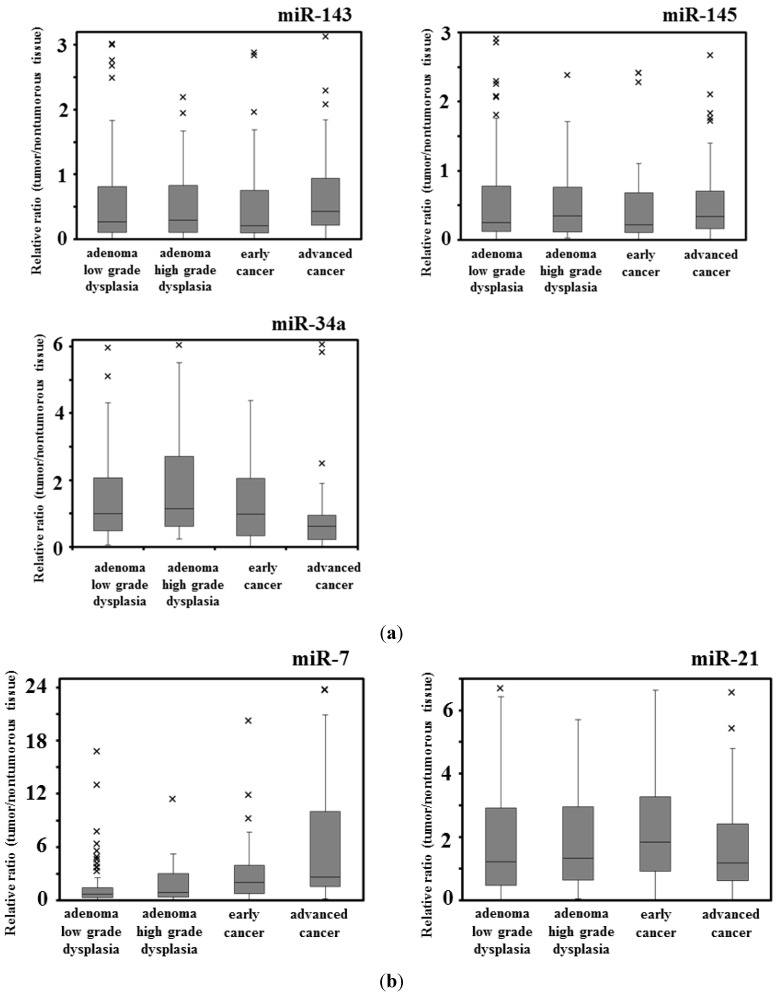
Box-and-whisker plots of miRNA expression in colorectal tumors. (**a**) Down-regulated miRNAs (miR-143, -145, and -34a); and (**b**) Up-regulated miRNAs (miR-7 and -21). ×: outlier of miRNA expression.

The expression levels of miRs-143 and -145 were down-regulated evenly in almost or more than 70% of both cancers and adenomas. Importantly, both miRNAs were down-regulated concomitantly in most of both cases, because they are known to be transactivated as the same primary miRNA, NCR143/145 [12] (Table 1, Figure 2a). On the other hand, the up-regulation of miR-7 was frequent, being especially significant in the cancers (Table 1, Figure 2b). The expression level of miR-34a was also significantly down-regulated more frequently in cancers than in adenomas (Table 1, Figure 2a). The addition of down-regulation of miR-34a to the decreased expression of miR-143 and -145 may be a trigger for the promotion of cancer development. Notably, as to miR-7 in cancer development, the increased expression of miR-7 may have affected the cancer development independently of the down-regulation of miR-143 and -145 (Figure 2b). The frequency of the up-regulation of miR-21 was almost the same between cancers and adenomas (Table 1, Figure 2b).

### 2.2. Expression of miR -143, -145, -7, -34a, and -21 in Human Colorectal Cancers (Early vs. Advanced) and Adenomas (Low- vs. High-Grade Dysplasia)

Next, we analyzed the expression levels of these miRNAs in 44 tumor samples from early cancers and 91 samples from advanced cancers (Table 3, Figure 2). The expression levels of miRs-143, -145, and -34a were decreased frequently in both early and advanced cancers. In contrast, the up-regulation of miR-21 was more frequent in early cancers than in advanced ones. On the other hand, the expression level of miR-7 was increased especially in advanced cancers compared with that in the early cancers.

As to the adenomas, we analyzed the expression levels of these miRNAs in 99 adenoma samples with low-grade dysplasia and in 40 with the high-grade type (Table 3, Figure 2). The expression levels of miR-143 and -145 were down-regulated frequently in both low- and high-grade dysplasias. However, miR-7 was up-regulated more frequently in high-grade dysplasia than in low-grade dysplasia. The frequencies of up-regulation of miR-21 and down-regulation of miR-34a were almost the same between low- and high-grade dysplastic adenomas.

**Table 3:** Expression of miR-143, -145, -7, -34a, and -21 in human colorectal cancer (early or advanced cancer) and adenoma tissues (low- or high-grade dysplasia).

Colorectal Tumor	*n*	miR-143 ↓	miR-145 ↓	miR-7 ↑	miR-21 ↑	miR-34a ↓
colorectal cancer						
early cancer depth M, SM	44	32	32	25	27	21
advanced cancer depth MP-SI, A	91	63	67	69	37	53
*p* value		0.6767	0.9118	0.0244	0.0240	0.2499
colorectal adenoma						
low grade dysplasia	99	67	68	23	41	37
high grade dysplasia	40	27	27	18	18	11
*p* value		0.9839	0.8917	0.0108	0.6986	0.2677

Down arrow: down-regualted; Up arrow: up-regulated. The red font means that there is a statistical significance.

### 2.3. Relationship between the Expression of Onco-Related miRNAs and Endoscopic Appearance of Colorectal Tumors

**Figure 3:**
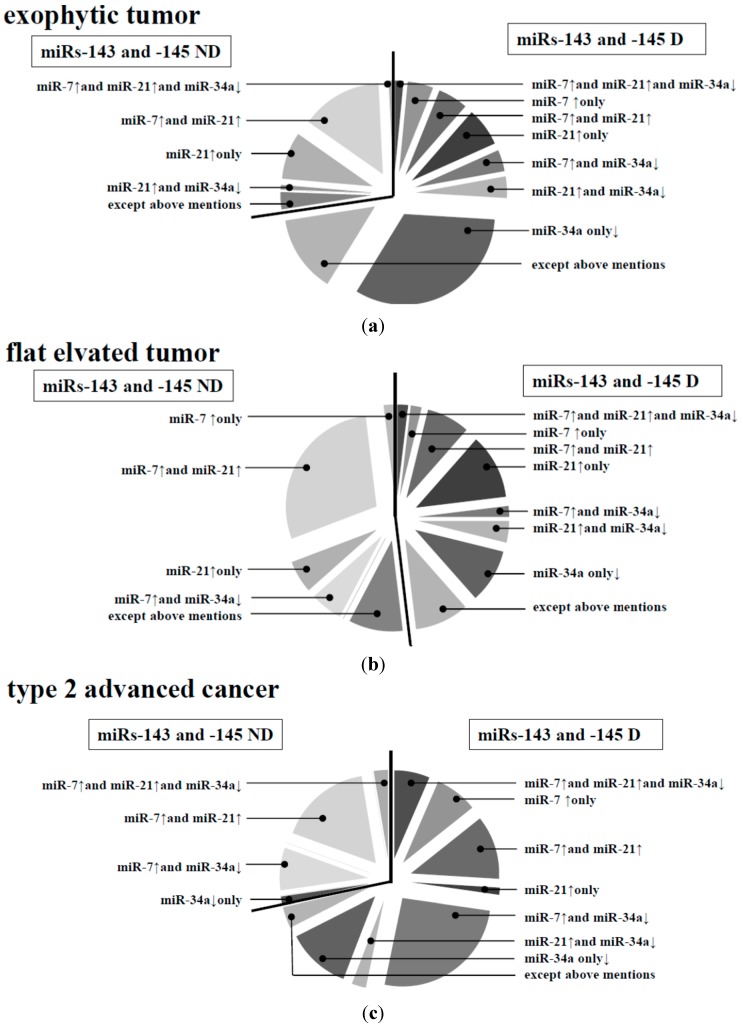
Expression profiles of miRs-7, -34a, and -21 with or without down-regulation of miRs-143 and -145 in human colorectal tumors. (**a**) Exophytic type; (**b**) Flat elevated type; and (**c**) Type 2 advanced cancer. D, down-regulated; ND, not down-regulated. Except above mentions: miR-7 and miR-21 are not up-regulated, and miR-34a is not down-regulated.

Based on these results, we focused on the difference in the forms of growth that appeared in early cancers and adenomas. We analyzed the expression levels of the miRNAs in 131 samples of exophytic tumors and in 52 samples of flat elevated tumors (Table 4, Figure 3). Basically, the expression levels of miR-143 and -145 were significantly down-regulated in more exophytic tumors than in flat elevated tumors. In both types, miR-34a was additionally down-regulated. On the other hand, for flat elevated tumors, there were two types: a group with down-regulated miR-143 and -145 and one with up-regulated miR-7 and/or -21 (Figure 3). The increased levels of miR-7 and -21 occurred independently of the down-regulated miR-143 and -145 in the flat elevated tumors. Most cases of up-regulated miR-7 were accompanied by miR-21 up-regulation, but not with miR-143 and -145 down-regulation (Figure 3). These findings were typical in adenomas, but not in early cancers (data not shown). Large flat elevated tumors (>10 mm) or so-called laterally spreading tumors (LSTs) were classified as granular type (LST-G) or non-granular type (LST-NG) according to their endoscopic appearance. When we analyzed the expression levels of the miRNAs in both of them (Table 5), interestingly, the expression level of miR-21 was found to be significantly up-regulated in more LST-NG than in LST-G samples. Importantly, there were only six cases of type 1 advanced colorectal cancer; however, there was a significant difference in the expression profile of miR-7 between type 1 and type 2 cancers (Table 6). When we examined the expression levels of these miRNAs of type 2 in comparison with those of exophytic tumors or flat elevated tumors (Table 6), the profiles of type 2 and exophytic tumors were very similar to each other in colorectal cancers and adenomas. In contrast, the expression patterns of type 2 and flat elevated tumors were significantly different. These data on miRNA profiles strongly suggested that the type 2 cancer most likely originated from the exophytic type, perhaps the adenoma-carcinoma sequence.

### 2.4. Expression of miR-7 in Human Colorectal Cancer Cell Lines

**Figure 4:**
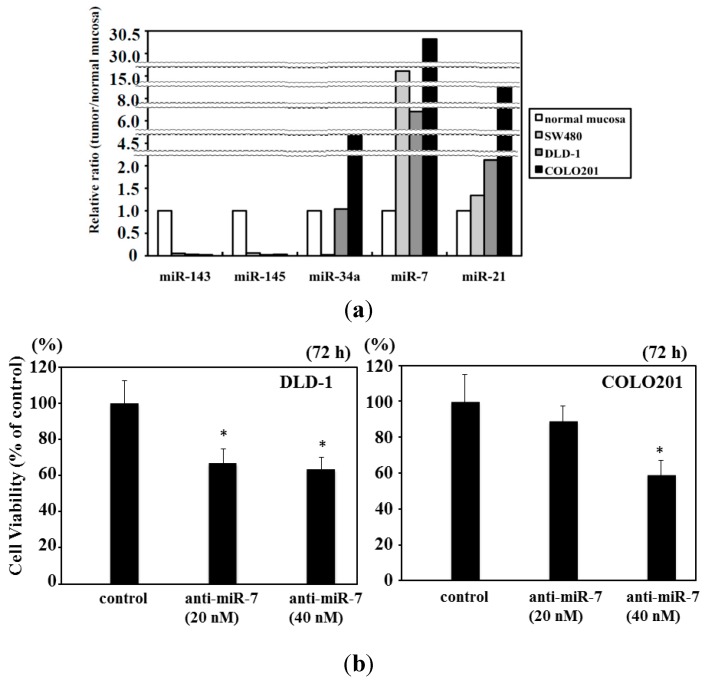
Expression of miR-143, -145, -7, -34a, and -21 in human colon cancer cell lines. (**a**) Expression of the indicated miRNAs in various human colon cancer cell lines. The relative expression level in the cells transfected with control miRNA is indicated as “1”; and (**b**) Effect of ectopic expression of anti-miR-7 on proliferation of human colon DLD-1 and COLO201 cells. Anti-miR-7 was used for the transfection of the cells (20–40 nM), which was achieved by using cationic liposomes. The effects manifested by the introduction of the anti-miR-7 were assayed at 72 h after the transfection. The viable cell number of control cells at 72 h after the transfection is indicated as “100%”. Wave line: omission of vertical axis. *: There is statistical significant compare with normal control.

In our data, the frequency of up-regulated miR-7 increased with the progression of the clinical stages; and up-regulation rose faster at the stage of the flat elevated type than at that of the exophytic types. In our *in vitro* study, miR-7 was up-regulated in colorectal cancer cell lines SW480, DLD-1, and COLO201 (Figure 4a); and cell growth was suppressed by transfection of the cells with anti-miR-7 (Figure 4b). No obvious signs of cell death such as apoptosis or autophagic cell death were found in the anti-miR-7 transfected cells. Thus, miR-7 could function as an oncogenic miRNA in colorectal cancer cells.

Clearly, different expression profiles of miRNAs in addition to the down-regulated miR-143 and -145 were found. It should be noted that the expression patterns of miR-143, -145, -34a, -7, and -21 contributed to the pathogenesis by affecting the morphological characteristics of the colon tumors.

**Table 4:** Expression of miR-143, -145, -7, -34a, and -21 in human colorectal adenoma and early cancer tissues (exophytic and flat elevated types).

Colorectal Tumor	miR-143 ↓		miR-145 ↓		miR-7 ↑		miR-21 ↑		miR-34a ↓	
	*n*	Average Fold	*n*	Average Fold	*n*	Average Fold	*n*	Average Fold	*n*	Average Fold
exophytic type (*n* = 131)	99	0.50	102	0.53	40	3.80	55	1.80	57	1.70
flat elevated type (*n* = 52)	27	0.99	25	1.02	26	3.71	31	3.47	12	2.09
*p* value	0.0018	−	<0.0001	−	0.0134	−	0.0311	−	0.0101	−

Down arrow: down-regualted; Up arrow: up-regulated. The red font means that there is a statistical significance.

**Table 5:** Expression of miR-143, -145, -7, -34a, and -21 in human colorectal adenoma and early cancer tissues (laterally spreading tumor (LST): granular type (LST-G) and non-granular type (LST-NG)).

Colorectal Tumor Flat Elevated Type	miR-143 ↓		miR-145 ↓		miR-7 ↑		miR-21 ↑		miR-34a ↓	
	*n*	Average Fold	*n*	Average Fold	*n*	Average Fold	*n*	Average Fold	*n*	Average Fold
LST-G (*n* = 26)	15	0.78	13	0.77	12	2.96	13	2.89	6	2.35
LST-NG (*n* = 17)	8	1.18	8	1.33	11	5.77	14	5.15	2	2.13
*p* value	0.4943	−	0.8504	−	0.2331	−	0.0321	−	0.3038	−

Down arrow: down-regualted; Up arrow: up-regulated. The red font means that there is a statistical significance.

**Table 6:** Expression of miR-143, -145, -7, -34a, and -21 in human colorectal tumor tissues (advanced cancer types 1 and 2; exophytic tumor; flat elevated tumor).

Colorectal Tumor	*n*	miR-143 ↓	miR-145 ↓	miR-7 ↑	miR-21 ↑	miR-34a ↓
type 1 advanced colorectal cancer	6	6	6	2	2	4
type 2 advanced colorectal cancer	77	55	57	61	32	45
*p* value (type 1 *vs.* type 2)		0.1471	0.1800	0.0278	0.5237	0.5237
exophytic tumor	131	99	102	40	55	57
*p* value (type 2 *vs.* exophytic tumor)		0.5104	0.5290	<0.0001	0.9520	0.0375
flat elevated tumor	52	27	25	26	31	12
*p* value (type 2 *vs.* flat elevated tumor)		0.0239	0.0027	0.0005	0.0442	<0.0001

Down arrow: down-regualted; Up arrow: up-regulated. The red font means that there is a statistical significance.

## 3. Discussion

The carcinogenesis of time-dependent colorectal tumor development has been well-established as the adenoma-carcinoma sequence theory. According to this theory, no matter whether the tumors are hereditary colon cancers or non-hereditary ones, they are thought to become malignant in the following manner: normal epithelium → adenoma → carcinoma → metastasis. In addition, aberrations in plural oncogenes and tumor suppressor genes are involved in this sequence of molecular events [31,32,33]. On the other hand, another theory for carcinogenesis has been proposed, *i.e*., the *de novo* theory [21,34,35]. According to this theory, the flat elevated tumors are pathologically diagnosed as colorectal cancer, having developed without the adenoma step [35]. Moreover, the flat elevated colorectal neoplasms exhibit more malignant characteristics than exophytic neoplasms [36]. However, the *Ki-Ras* gene mutation is not more frequent in flat elevated tumors compared with its frequency in exophytic tumors [22,28,29]. Especially, the frequency of this mutation in LST-NG is lower than that in LST-G [28,29]. Thus, the relationship between the endoscopic appearance and gene aberration is now highlighted by these two types of flat elevated tumor. So far, there has been no report concerning the relation between endoscopic appearance and the expression profile of miRNAs for discriminating the *de novo* type from the adenoma-carcinoma sequence.

The inappropriate expression of miRNAs is closely associated with cancer development. We examined the expression of anti-oncogenic and oncogenic miRNAs that were aberrantly expressed mainly in colon tumors. The combined decreased expressions of miR-143 and -145 are frequently observed in most cancers and even in colon adenomas, and these miRNAs function as tumor suppressors [10,11,12,13,37]. Also, p53 transactivates miR-145, which then targets *c-myc* at the translational level [38]. On the other hand, miR-143 targets *Erk5*, which transactivates *c-myc* downstream [39]. Therefore, the coincidental down-regulation by both miRNAs results in the up-regulation of c-myc, which would be an essential event in colon tumor development [40,41]. Our data also suggests that the frequency of the down-regulation of both miRs-143 and -145 was significantly higher in exophytic types than in the flat elevated types. The expression level of miR-34a is frequently down-regulated in colorectal cancers [13,14,15]. The targets of miR-34a are SIRT-1 (sirtuin-1) [14], SND1 (Staphylococcal nuclease homology domain containing) [15], and a component of the positive-feedback loop of the p53 tumor suppressor network [15]. Our data suggested that the down-regulation of miR-34a in adenomas was significantly greater in the samples of the exophytic type than in those of the flat elevated type. In contrast, the expression level of miR-7 is up-regulated in advanced colorectal cancers and cancer cell lines [13] and in the stool from a patient with colorectal cancer [19]. Thus, it is to be noted that miR-7 functioned as an oncogenic miRNA in colon cancer, because it was up-regulated in several colon cancer cells and the transfection of human colon cancer DLD-1 cells with anti-miR-7 suppressed proliferation of these cells (this study). On the other hand, there is a report that the expression level of miR-7 is down-regulated in advanced colorectal cancer cell lines [20]. In our data, we compared the normal mucosa paired with colorectal cancer; and the frequency of miR-7 up-regulation increased with the progression of the stages. However, there was no relationship between the frequency of miR-7 up-regulation and the size of tumor. The frequency of miR-7 up-regulation was higher at the stage of the flat elevated type than at that of the exophytic one. Interestingly, the expression level of miR-21 is up-regulated in colorectal tumors of the flat elevated type along with the down-regulation of miR-143 and -145 [13,16,17,18]. The target of miR-21 includes PTEN (phosphatase and tensin homologue) [17] and PDCD4 (programmed cell death 4) [18], which are closely related to tumorigenesis. MiR-21 may have contributed to the triggering of carcinogenesis (Table 3, Figure 2b), especially in the case of the flat elevated type (Table 3, Figure 3); and it may have contributed to the triggering of malignant transformation without the down-regulation of miRs-143 and -145. MiR-7 could contribute to the malignant transformation of both exophytic and flat elevated types.

There is a report that flat elevated colorectal neoplasms exhibit more malignant phenotypes than exophytic neoplasms [36]. LST-NG generally displays more malignant characteristics than LST-G [27]. It is useful to discriminate the flat elevated colorectal neoplasms, especially LST-NG, at colonoscopy. It is often difficult to find flat elevated colorectal neoplasms as opposed to exophytic neoplasms. In our data, the patterns of miRNA expression between exophytic and flat elevated neoplasms were not similar to each other. As to the flat elevated type, there were some cases in which up-regulation of miR-7 and -21 occurred without down-regulation of miR-143 and -145. In particular, it should be noted that the frequency of miR-21 up-regulation was higher in LST-NG than in LST-G, which may indicate that the former was more malignant than the latter. Furthermore, our data suggested that type 2 advanced colorectal cancers could have originated from the exophytic type of the adenoma-carcinoma sequence based on the data for miRNA expression profiles, because the miRNA expression profile of type 2 cancers was similar to that of the exophytic type. The expression profiles of miRNAs tested suggest that there were different pathways to exophytic and flat elevated tumors, respectively, as in the mutation profiles of their genomic data.

As to the quantitative miRNA assays, more examination methods using other platforms such as sequencing, miRNA array, and other RT-qPCR methods are needed to further validate miRNA expression [42]. While the down-regulation of miRs-143, -145, and -34a and the up-regulation of miRs-7 and -21 appear to be clearly discriminated in each category of colorectal tumors with the current method used [11,13,43], more data will be needed to further validate these miRNAs for application as biomarkers that discriminate the characteristics of morphological appearance.

**Figure 5:**
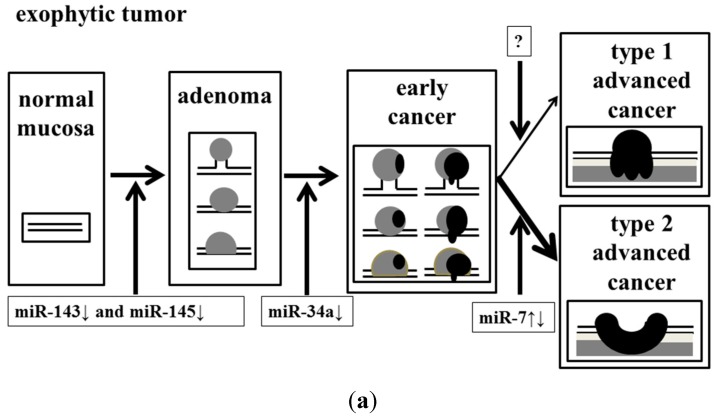

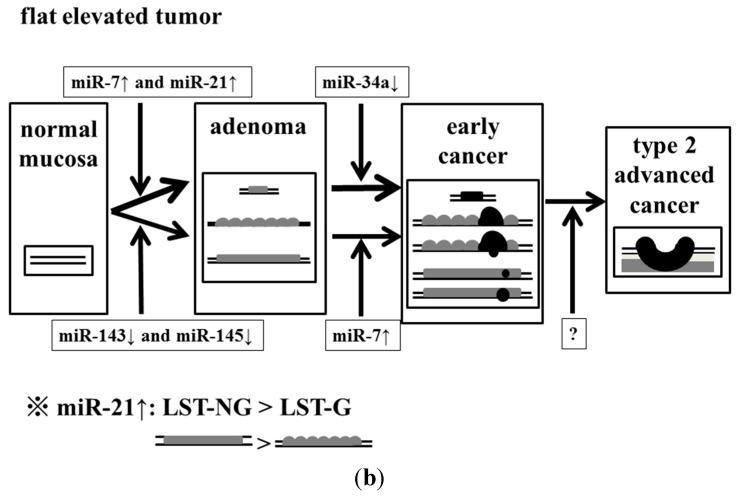
Effect of accumulation of aberrant miRNA expression on the process of malignant transformation in exophytic and flat elevated tumors. (**a**) Exophytic tumor; and (**b**) Flat elevated tumor.

In conclusion, we determined that the dysregulation of these miRNAs might have affected the endoscopic appearance of colorectal tumors (Figure 5).

## 4. Experimental Section

### 4.1. Case of Colorectal Tumors, Diagnosis of Colonoscopy

All human samples were obtained in the fresh state from patients who had undergone a direct biopsy for diagnosis or surgery for resection of colorectal tumors at Fujita Health University Hospital (Aichi, Japan), Saiseikai Ibaraki hospital (Osaka, Japan), Osaka Medical College Hospital (Osaka, Japan) or Kyoritsu General Hospital (Aichi, Japan) between 2002 and 2013. All cases of this study were diagnosed by high-definition endoscopy. There was no sample of fresh frozen sample or formaline-fixed, paraffin-embedded (FFPE) sample used in this study. Most cases of adenomas and early cancers were diagnosed by their pit pattern and narrow band imaging by using magnifying endoscopy. Most cases of flat elevated tumors and more than half of the exophytic tumors were those of patients who had undergone endoscopic membrane resection (EMR) or endoscopic submucosal dissection (ESD). More than half of the exophytic tumors were obtained from patients in whom EMR or endoscopic polypectomy was performed by snaring. Especially, all LSTs were diagnosed by using magnifying endoscopy. Final endoscopic diagnosis of colorectal tumors was determined by 2 endoscopists who belong to the fellow of the Japanese Society of Gastroenterology and are board-certified fellows of the Japan Gastroenterological Endoscopy Society (Figure 1a,b). Two pathologists diagnosed each sample based on the Japanese Classification of Colorectal Carcinoma (8th edition) [30]. Cases in which endoscopic images of colorectal tumor morphology were not good and/or the diagnosis of the pathologist differed from that of the endoscopists, were excluded from this study. The pathological specimens for which there was a suspicion of early colorectal cancer and/or LST were categorized after a discussion between pathologists and endoscopists. Informed consent in writing was obtained from each patient. And the protocol was approved by the Ethics Committee of Fujita Health University Hospital.

### 4.2. Cell Lines, Cultures, Transfection with Anti-miR-7

Human colon cancer cell lines SW480 (American Type Culture Collection (ATCC)^®^, CCL-228™; Manassas, VA, USA), DLD-1 (ATCC^®^, CCL-221™), and COLO201 (ATCC^®^, CCL-224™) were grown in RPMI-1640 medium supplemented with 10% (*v*/*v*) heat-inactivated FBS (Sigma, St. Louis, MO, USA) and 2 mM l-glutamine under an atmosphere of 95% air and 5% CO_2_ at 37 °C. The number of viable cells was determined by the trypan-blue dye exclusion test. DLD-1 or COLO201 cells were seeded in 6-well plates at a concentration of 1 × 10^5^–1.5 × 10^5^/well (25%–35% confluence) the day before transfection. An inhibitor of miR-7 (anti-miR-7, mirVanaTM miRNA inhibitor; Ambion, Austin, TX, USA) was used for the transfection of the cells (20–40 nM), for which transfection was achieved by using cationic liposomes, LipofectamineRNAiMAX (Invitrogen, Carlsbad, CA, USA), according to the manufacturer’s Lipofection protocol. The transfection efficiency was evaluated by the transfection of the cells with a duplex siRNA-FITC (Dharmacon, Lafayette, CO, USA) and was found to be more than 80% for DLD-1 or COLO201 cells. Non-specific control miRNA (NS, 57% GC content; Dharmacon) was used as a control for non-specific effects. The effects manifested by the introduction of the anti-miR-7 used in this study into the cells were assayed at 72 h after the transfection. The viable cell number of control cells at 72 h after the transfection was taken as “100%”.

### 4.3. RNA Isolation, Real-Time PCR of microRNA

Total RNA was isolated from the tissues by use of TRIzol containing phenol/guanidium isothiocyanate and treatment with DNase I. In order to examine the expression levels of miRNAs in detail, we performed TaqMan^®^ MicroRNA Assays using a real-time PCR apparatus (Life Technologies, Grand Island, NY, USA) [13,37,43]. We examined the expression levels of tumor miRNAs compared with those of the paired normal samples in a blinded fashion. The threshold cycle (*C*_t_) is defined as the fractional cycle number at which the fluorescence passes a fixed threshold. The range of *C*_t_ values of these miRNAs in colorectal cancer was from 18 to 40. In our data, we judged that there was no miRNA expression over 28 cycles, because there were miRNA molecules less than 50 copies per 1 mL in *C*_t_ value 28. The levels of miRNAs in each tissue were measured and normalized to those of U6, which was used as an internal control [11,13]. The relative expression levels were calculated by the ΔΔ*C*_t_ method. The relative expression level in normal tissue was indicated as “1”. The expression levels in tumors were designated as down-regulated when the fold change from the expression in the non-tumorous tissue was 0.67 or as up-regulated when the fold change from the expression in the non-tumorous tissue was 1.50, as determined from the results of linear discriminant analysis of miRNA expression patterns from 274 pairs of colon tumors and non-tumorous tissues. The tumor/non-tumor ratio of each miRNA expression in the samples was determined. The tumor/non-tumor ratio of each miRNA expression in the samples was expressed by use of Box-and-whisker plots.

### 4.4. Statistical Analysis

Each examination was performed in triplicate. In experiments on clinical samples, the expression levels >1.5 were designated as up-regulation and those <0.67 as down-regulation, for which fold changes were obtained from the results of linear discriminant analysis of miRs-143 and -145 expression patterns from 135 pairs of colon tumors and non-tumorous tissues. Statistical differences of miRNA levels were evaluated by using Pearson’s χ^2^ test or Fisher’s extract test for differences between 2 groups. A *p*-value of 0.05 was considered to be significant. All calculations were performed by using software JMP (version 5.1; SAS Inc., Cary, NC, USA).

## 5. Conclusions

In this study, we mainly analyzed the relationship between endoscopic appearance and miRNA profile of colorectal tumors. The frequency of the down-regulation of both miRs-143 and -145 was significantly higher in exophytic than in the flat elevated types of tumors. The down-regulation of miR-34a in adenomas was significantly greater in the samples of the exophytic type than in those of the flat elevated type. MiR-7 functioned as an oncogenic miRNA in colon cancer. The frequency of miR-7 up-regulation was higher at the stage of the flat elevated type than at that of the exophytic one. The expression level of miR-21 was up-regulated in colorectal tumors of the flat elevated type along with the down-regulation of miR-143 and -145. The frequency of miR-21 up-regulation was higher in LST-NG than in LST-G. Furthermore, our data suggested that type 2 advanced colorectal cancers could have originated from the exophytic type of the adenoma-carcinoma sequence based on our data for miRNA expression profiles. We determined that the dysregulation of these miRNAs might have affected the endoscopic appearance of colorectal tumors (Figure 5).
